# Overcoming immunosuppression in cancer: how ketogenic diets boost immune checkpoint blockade

**DOI:** 10.1007/s00262-024-03867-3

**Published:** 2024-11-13

**Authors:** Victoria E. Stefan, Daniela D. Weber, Roland Lang, Barbara Kofler

**Affiliations:** 1 Research Program for Receptor Biochemistry and Tumor Metabolism, Department of Pediatrics, University Hospital of the Paracelsus Medical University, Salzburg, Austria; 2 Department of Biosciences and Medical Biology, University of Salzburg, Salzburg, Austria; 3 Department of Dermatology and Allergology, University Hospital of the Paracelsus Medical University, Salzburg, Austria

**Keywords:** Ketogenic diet, Cancer, Immunometabolism, Anti-tumor immune response, Immune checkpoint blockade

## Abstract

Immune checkpoint blockade (ICB) is now part of the standard of care in the treatment of many forms of cancer, yet it lacks efficacy in some patients, necessitating adjunct therapies to support the anti-tumor immune response. Ketogenic diets (KDs), i.e., high-fat low-carbohydrate diets, have been shown to have antiproliferative and immunomodulatory effects in various preclinical cancer studies. Here, we review current knowledge of the complex interplay of KDs and the anti-tumor immune response in the context of ICB therapy, to update our understanding of diet-induced immunometabolic reprogramming in cancer. Preclinical cancer studies have revealed increased activation of and infiltration by tumor-fighting immune cells, especially CD8+ T cells, but also M1 macrophages and natural killer cells, in response to a KD regimen. In contrast, immune-suppressive cells such as regulatory CD4+ T lymphocytes, M2 macrophages, and myeloid-derived suppressor cells were reported to be decreased or largely unaffected in tumors of KD-fed mice. KDs also showed synergism with ICB therapy in several preclinical tumor studies. The observed effects are ascribed to the ability of KDs to improve immune cell infiltration and induce downregulation of immune-inhibitory processes, thus creating a more immunogenic tumor microenvironment. The studies reviewed herein show that altering the metabolic composition of the tumor microenvironment by a KD can boost the anti-tumor immune response and diminish even immunotherapy-resistant as well as immunologically "cold" tumors. However, the exact underlying mechanisms remain to be elucidated, requiring further studies before KDs can be successfully implemented as an adjunct tumor therapy to improve survival rates for cancer patients.

## Background

Despite impressive advancements in cancer therapy due to novel treatment approaches such as immune checkpoint blockade (ICB), cancer patients still experience relapse, severe side effects, and poor quality of life. Established therapies frequently fail to induce sufficient and sustainable tumor regression. However, novel adjuvant therapies, immunotherapy, and extensive monitoring of the anti-tumor immune response are helping to mitigate these hallmarks of cancer.

A major challenge is that cancer cells often induce an immune-suppressive tumor microenvironment (TME) which impedes the body’s anti-tumor immunity [1]. These so-called immunologically "cold" tumors require therapeutic reprogramming of the patient’s immune system to recognize and eliminate cancer cells [2]. Currently, the predominant approach used to switch on a dormant anti-tumor immune response is immunotherapy [3]. ICB, which involves inhibition of programmed cell death protein 1 (PD-1)/ programmed cell death ligand 1 (PDL-1), cytotoxic T lymphocyte-associated protein 4 (CTLA-4), and lymphocyte-activation gene 3 (LAG-3), promotes a robust CD8+ T cell response against cancer cells, the first step toward sustainable tumor regression [4]. However, direct inhibition of immune cells by malignant cells is not the only obstacle faced by cancer therapy. Solid tumors, especially, are poorly penetrated by lymphocytes, for multiple reasons, including absence of neoantigens, low expression of major histocompatibility complex I (MHC-I), and secretion of inhibitory cytokines such as transforming growth factor beta (TGF-β) and interleukin 10 (IL-10) [2]. Consequently, tumor relapse remains frequent, and both response and survival rates continue to be poor in a plethora of cancer entities, necessitating novel treatment approaches.

Emerging adjunct therapies are targeting the altered metabolism of cancer cells, which includes both long-known and more recently added hallmarks of cancer [1, 5]. The most established of these metabolic characteristics is described as the Warburg effect. In order to meet the high demands in energy and biomass, concomitant with a high proliferation rate, tumor cells shift their metabolism from oxidative phosphorylation (OXPHOS) toward aerobic glycolysis. Therefore, even upon normal nutrient and oxygen availability, glucose is metabolized to pyruvate and further to lactate, allowing more rapid production of both ATP and biomass [6].

Consequently, depriving cancer cells of glucose renders them vulnerable and can therefore mitigate tumor growth [7]. This can be achieved, for example, with a high-fat low-carbohydrate ketogenic diet (KD) with adequate protein content. The low-carbohydrate content triggers the production of ketone bodies (KBs), in particular beta-hydroxybutyrate (BHB) and acetoacetate (AcAc), from fatty acids in the liver, providing an alternative energy source for extrahepatic tissues [8].

Recently, numerous preclinical studies have reported anti-tumor effects of KDs, thus supporting their use as an adjunct metabolic cancer therapy [9, 10]. Evidence of antiproliferative effects of KDs in cancer patients, however, remains scarce, while an increase in quality of life has been reported in several clinical studies [9, 11]. Since under a KD regimen, the energy for normal cells is supplied by KBs, KDs almost exclusively target cancer cells. A variety of cancer entities cannot use KBs because the cells lack the required enzymes and have dysfunctional mitochondria [12]. However, the cancer-starvation hypothesis has recently been questioned, since we and others have observed that blood glucose levels remain unaffected upon a KD regimen in some preclinical studies [9, 13–16]. Therefore, glucose deprivation may not be the primary or sole cause of the observed anti-tumor effect of KDs. Rather other factors, for example alterations of the immune response, reduced insulin signaling, the levels of reactive oxygen species, the metabolic profile of the TME, the epigenome, and the microbiome, as well as KB-induced signaling pathways, could contribute to KD-mediated attenuation of tumor growth [9, 12, 17].

Recent findings also implicate immunometabolic reprogramming in the anti-tumor effect of dietary interventions [18]. However, evidence of changes in the TME induced by a KD remains scarce. Therefore, in this review, we summarize the results of existing studies of the interaction of KDs and anti-tumor immunity. Furthermore, we aim to improve understanding of this interaction and to highlight possible implications of KDs as an adjunct to ICB.

## KD and the anti-tumor immune response

The mechanisms known to underlie KD treatment efficacy have recently been expanded from purely metabolic to include both epigenetic and immune-modulatory activities [19]. The effect of KDs on the anti-tumor immune response, however, remains to be fully elucidated. To date, relatively few oncological KD studies have investigated immunological aspects of the metabolic treatment [9]: seven preclinical studies have analyzed the effect of KD on immune cells in the TME of glioma [13, 20], pancreatic cancer [21], and colon cancer [22–25] (Table 1). Notably, all of these studies also described a reduction in tumor growth and/or an increase in survival of tumor-bearing mice, pointing toward a possible connection between the observed survival benefits and immunological changes brought on by a KD.

**Table 1:** Overview of preclinical studies reporting an effect of KD on the anti-tumor immune response

Tumor type	Mouse model/strain	Ketogenic regimen	ICB	Glucose and ketone levels	Effect on treatment outcome	Effect on cancer cells	Effect on TME	Ref.
Glioma	Orthotopic glioblastoma C57BL/6	4:1 KD	–	↑ BHB, ↔ glucose	↑ survival	↓ proliferation	↓ CD4+ T cells, ↔ CD8+ T cells, ↔ Tregs, ↓ M1 macrophages, ↑ M2 macrophages, ↑ NK cells, ↓ MDSCs	[13]
	Orthotopic glioma C57BL/6	4:1 KD	–	↑ BHB, ↓ glucose	↑ survival	↓ proliferation, ↓ expression of PDL-1, CD86	↑ Th1 CD4+ T cells, ↔ CD8+ T cell numbe, r ↑ CD8+ T cell activity, ↔ Tregs, ↔ NK cell number, ↑ NK cell activity	[20]
Pancreatic Cancer	s.c. pancreatic cancer C57BL/6	6:1 KD	–	↑ BHB, ↓ glucose	↑ survival	↓ proliferation	↑ CD8+ T cells, ↑ CD4+ T cells, ↓ Tregs, ↓ MDSCs	[21]
Colon Cancer	s.c. colon cancer BALB/c	4:1 KD	–	↑ BHB, ↓ glucose, ↓ intratumoral lactate	–	↓ proliferation	↑ M1 macrophages, ↓ M2 macrophages, ↑ NK cells, ↓ MDSCs	[23]
	s.c. colon cancer BALB/c	4:1 KD	–	↑ BHB, ↓ glucose	–	↓ proliferation, ↓ expression of PDL-1, CTLA-4	↑ Th1 CD4+ T cells, ↑ CD8+ T cell number and activity, ↔ Tregs, ↑ CCL5, CXCL9, CXCL10, CXCL11, ↑ NK cell activity	[24]
	s.c. colorectal cancer BALB/c and C57BL/6	6:1 KD	–	↑ BHB, ↓ glucose	–	↓ proliferation	↑ CD4+ T cells, ↑ CD8+ T cells, ↓ Tregs, ↑ M1 macrophages, ↓ M2 macrophages, ↑ NK cells, ↓ MDSCs	[22]
			Anti-PD-1		KD synergized with ICB, ↑ survival	↓ proliferation	↓ CXCL12 expression by CAFs, ↓ repression of immune cells	
	s.c. colon cancer BALB/c	6:1 KD	–	↓ glucose, ↓ insulin	↔ survival	↓ proliferation	↔ T cells	[25]
			Anti-CTLA-4		KD synergized with ICB, ↑ survival	↓ proliferation, ↑ antigen presentation	↑ CD8+ T cell number and activity, ↔ CD4+ T cells, ↔ Tregs, ↑ macrophages, ↔ MDSCs	
Prostate Cancer	s.c. prostate cancer C57BL/6 with acquired ICB resistance	3:1 cyclic KD or 1,3-butanediol suppl.	anti-PD-1, anti-CTLA-4	↑ BHB	KD or BHB suppl. synergized with ICB, ↑ survival	↓ proliferation, ↑ MHC-I expression via HDACi	↑ CD8+ T cell number and activity, ↑ antigen-presenting monocytes, ↑ M1 macrophages, ↓ M2 macrophages, ↑ DCs, ↓ neutrophils, ↓ mesenchymal cells, ↑ antigen presentation and IFN-γ response genes in myeloid cells, ↑ cell-cell communications	[55]
Melanoma	s.c. melanoma C57BL/6	4:1 KD or BHB suppl.	anti-PD-1 and anti-CTLA-4	↑ BHB	KD or BHB suppl. synergized with ICB, ↑ survival	↓ proliferation	–	[33]
Renal Cell Carcinoma	orthotopic renal cancer BALB/c	intermittent 4:1 KD or BHB suppl.	anti-PD-1 and anti-CTLA-4	↑ BHB	KD or BHB suppl. synergized with ICB, ↑ survival	↓ proliferation	–	
Lung Cancer	orthotopic lung cancer C57BL/6 with primary ICB resistance	BHB suppl.	anti-PD-1 and anti-CTLA-4	↑ BHB	BHB suppl. synergized with ICB, ↑ survival	↓ proliferation	–	

↑: increased, ↓: decreased, ↔ : not altered, BHB: beta-hydroxybutyrate, CAFs: cancer-associated fibroblasts, CCL: C–C motif chemokine, CTLA-4: cytotoxic T lymphocyte-associated protein 4, CXCL: C-X-C motif chemokine, DCs: dendritic cells, HDACi: histone deacetylase inhibition, ICB: immune checkpoint blockade, IFN-γ: interferon gamma, KD: ketogenic diet (specified by ratio of fat [g] to the sum of proteins [g] and carbohydrates [g]), MDSCs: myeloid-derived suppressor cells, MHC-I: major histocompatibility complex 1, NK cells: natural killer cells, PD-1: programmed cell death protein 1, PDL-1: programmed cell death ligand 1, s.c.: subcutaneous, suppl.: supplementation

### T cells

T cells play a pivotal role in tumor immune surveillance. They are generally perceived as highly glycolytic, especially when activated. Therefore, T cells compete with cancer cells for nutrients, particularly for glucose but also for glutamine and fatty acids, all of which are essential for both tumor cell growth and effector T cell function [26, 27]. T lymphocytes, however, also appear to have the ability to draw energy from KBs via ketolysis [28], unlike most cancer cells. Hence, a KD could give T cells a competitive advantage over tumor cells in the context of a TME with a limited glucose supply, by providing an alternative energy source.

#### CD8+ T cells

In vitro studies have indeed shown that treatment of primary human peripheral blood mononuclear cells (PBMCs) with BHB augments T cell proliferation and activity [29]. Cytotoxic CD8+ T cells, especially, seem to thrive in the presence of KBs through epigenetic remodeling and even prefer BHB over glucose to fuel the tricarboxylic acid (TCA) cycle [28]. Accordingly, one of the most reported immune-modulatory effects of KDs in preclinical cancer studies is a superior CD8+ T cell-mediated anti-tumor response.

Tumor-bearing mice administered a KD have shown qualitative and/or quantitative increases in CD8+ tumor-infiltrating lymphocytes (TILs) in most preclinical studies [20–22, 24]. One study of murine colon cancer indicated no significant alterations in CD8+ TILs [25]. Still, tumor infiltration by CD8+ T cells was enhanced by a KD in two other studies of murine colon cancer [22, 24] and one of pancreatic cancer [21]. Sun et al*.* ascribed the observed increase in cytotoxic TILs to the upregulation of T cell-recruiting chemokines such as C–C motif chemokine 5 (CCL5), C-X-C motif chemokine 9 (CXCL9), CXCL10, and CXCL11 [24].

In studies of murine glioma, the amount of CD8+ lymphocytes detected in tumors was not different between KD- and standard diet-fed mice [13, 20]. However, CD8+ TILs that have been detected in glioma and colon cancer showed increased activation and cytotoxicity, as indicated by elevated levels of interferon gamma (IFN-γ), tumor necrosis factor alpha (TNF-α), and IL-2 protein [20] or RNA synthesis [24]. In glioma, the increase in T cell activation markers was concomitant with reduced levels of the exhaustion markers PD-1 and CTLA-4 involved in T cell suppression; the KD also decreased the expression of the respective ligand on the tumor cells, PDL-1, thereby hampering tumor-induced T cell inhibition [20].

Increased activity of T cells could also be explained, at least in part, by a KD-induced reduction of intratumoral lactate [23, 30], which is known to inhibit cytotoxic T cell function [31] and impede immunotherapy efficacy [32]. However, a murine pancreatic cancer model revealed that the effects of a KD on tumor growth were less pronounced in comparison with diminished lactate dehydrogenase A, but resulted in comparable changes in immune cell infiltration [21].

In summary, KDs contribute to the alleviation of CD8+ T cell suppression and ameliorate T cell function in the TME. Of note, the survival benefit of KDs observed in murine tumor studies was diminished upon deletion of CD8+ T cells [20, 33]. However, several studies using athymic mice, which lack functional T lymphocytes, still demonstrated an anti-tumor effect of a KD [9]. In another study directly comparing immune-compromised (athymic) and immune-competent tumor-bearing mice, KD-mediated anti-tumor activity was observed in both models. The beneficial effect was, however, notably enhanced in the presence of T cells, highlighting the impact of KD on adaptive immunity and its support of a more efficient and more durable anti-tumor response [34].

#### CD4+ T cells

Similarly to CD8+ T cells, CD4+ T cells have also been shown to be affected by KDs. In mouse models of glioma as well as colon and pancreatic cancer, elevated levels of CD4+ TILs were reported in KD compared to standard diet-fed mice [20–22, 24], whereas the number of CD4+ TILs remained unchanged in another murine colon cancer model [25]. Interestingly, a skewing of T cell differentiation toward tumor-killing Th1 rather than immune-suppressive Th2 cells has also been observed in KD groups [20, 24]. Therefore, a KD-boosted CD4+ T cell response could potentially contribute to the anti-tumor effects of KDs.

Regulatory CD4+ T cells (Tregs), on the other hand, seem to be scarcely affected by KDs. Tregs are recruited by the tumor to create an immune-suppressive environment and are unwanted players in the setting of cancer therapies, especially immunotherapies, as they are associated with poor outcomes and prognosis [35]. Therefore, targeting this immune-suppressive T cell subtype is an important aim in cancer immunotherapy. Considering the dependency of peripheral Tregs on fatty acid oxidation (FAO) rather than on glycolysis [26], gene expression analysis in PBMCs of healthy donors revealed that Tregs exhibit relatively high levels of *OXCT1*, the gene encoding the rate-limiting enzyme of ketolysis, 3-oxoacid CoA-transferase 1 (OXCT1; also known as succinyl-CoA-3-oxaloacid CoA transferase (SCOT)) [36], indicating their ability to metabolize KBs. Indeed, in vitro experiments with human samples revealed an increase in Tregs in the presence of KBs [29]. Thus, KDs are expected to support Treg function. However, Tregs in the TME are also highly capable of using lactate as their primary energy source [37], which further enhances Treg function by upregulating TGF-β [38]. Therefore, depletion of glucose and its concomitant metabolite lactate from the TME by a KD could contribute to a decrease in the immune-suppressive and tumor-promoting activities of Tregs.

Accordingly, a KD lowered both lactate and Treg levels in pancreatic cancer-bearing mice compared to a standard diet [21]. On the other hand, tumor-infiltrating Tregs were found to be scarcely affected by a KD in two murine studies of glioma [13, 20] and one of colon cancer [25]. The effect of a KD on Tregs in the TME therefore requires further investigation, although it may not be a primary mechanism for the anti-tumor immune response.

#### Memory T cell formation

A major obstacle on the path to preventing disease relapse is the lack of immunological memory formation in many tumors. Intriguingly, Yang et al. suggested that a KD can elicit a durable anti-tumor immune response, but only in the presence of T cells [34]. The ability of a KD to promote the formation of memory T cells could ensure a truly sustainable anti-tumor immune surveillance to prevent relapse in different cancer entities and therapy settings.

Indeed, the prevailing KD-associated metabolite BHB has been implicated in memory T cell formation. BHB is known to be a potent inhibitor of histone deacetylases (HDACs), which, among other histone modifications, target lysine beta-hydroxybutyrylation (Kbhb). Specifically, Kbhb of histone H3 at lysine 9 mediates the activation of a gene set involved in memory formation and maintenance. By preventing the removal of this specific modification by HDACs, BHB can induce CD8+ memory T cell formation [12]. Accordingly, Hirschberger and colleagues already showed that in vitro treatment of primary human PBMCs with BHB led to increased levels of CD4+ and CD8+ central and effector memory T cells [29]. Direct evidence of increased memory T cell formation upon a KD in tumor settings is however lacking.

Even among different cancer entities, the T cell-modulating effect of a KD has been consistently observed. There is strong evidence that a KD improves the T cell-mediated anti-tumor response by creating conditions favoring cytotoxic rather than regulatory T cells. We propose that the mechanisms underlying the effects of a KD on T cells in the TME are mediated by i) KB-sustained energy supply to T cells, which circumvents competition with cancer cells for other nutrients, ii) reduction of immune-suppressive/T cell-exhausting intratumoral lactate, and iii) a chemokine/cytokine pattern that promotes effector function, as well as the homing of cytotoxic T cells to the tumor.

### Macrophages

Macrophages are also key players in the TME, and their polarization state can impact cancer progression. Macrophages can polarize either in a classically activated, pro-inflammatory, tumor-killing M1 or an alternatively activated, immune-suppressive, and tumor-promoting M2 phenotype [39]. In the TME, tumor-associated macrophages (TAMs) are present and commonly perceived as pro-tumorigenic M2-like [40]. Depending on their polarization, macrophages exhibit different metabolic properties. M1 macrophages are highly glycolytic, while M2 rely more on OXPHOS and FAO [41]. This supports the assumption that a KD can promote the polarization of macrophages in the TME toward a pro-tumorigenic M2 state, thereby contributing to an immune-suppressive environment.

Accordingly, upregulation of pro-tumorigenic M2 macrophages was observed in a KD-administered mouse model of glioblastoma. The polarization of macrophages toward a more tumor-promoting phenotype (M2) in the TME of glioblastoma was assumed to be driven by fatty acids via PPAR-γ signaling [13]. KD-fed colon cancer-bearing mice, on the other hand, presented with elevated levels of M1- and reduced M2-like macrophages [22, 23]. However, in this latter model, application of a KD also downregulated CXCL12 secretion, which is associated with hampered infiltration by immune-suppressive macrophages [22].

Although reduction of tumor growth and an associated survival benefit of a KD were observed in all of the studies reviewed herein, the effect of a KD on macrophages seems to differ between cancer entities. This effect might depend on the metabolic profile of the specific cancer cells and of the TME, information which is largely lacking in these studies. Considering that activated M1 macrophages and TAMs are highly glycolytic, they are also fated to contest for glucose with cancer cells [41]. A KD can further decrease not only systemic but also intratumoral glucose availability [34]. Therefore, macrophages, especially classically activated ones, require alternative energy sources in the TME. Monaco et al.’s gene expression dataset revealed low levels of *OXCT1* in monocytic cells [36], implying low basal KB metabolism in macrophages. In vitro experiments, however, showed that macrophages, both alternatively and classically activated, prefer AcAc over glucose to fuel the TCA cycle [42]. KBs and fatty acids could therefore fuel macrophages in the TME. BHB and fatty acids, however, are also favored by M2 polarized macrophages [13, 43], indicating immune-suppressing effects of a KD. On the other hand, intratumoral lactate, which is a strong promotor of M2 polarization [44], was decreased by a KD [23, 30], suggesting another mechanism for how a KD could boost the M1-mediated innate anti-tumor immune response.

Of note, TAMs display different metabolic patterns compared to M1 and M2 macrophages in the periphery, necessitating thorough investigations of their glucose, fatty acid, and KB metabolism to allow for the elucidation of KD-induced changes in TAM polarization, infiltration, and activation, and their connection to KD-mediated survival benefits in cancer models. In summary, the effect of a KD on macrophages in the TME seems less ambiguous and less substantial than that on T cells but still requires further investigation.

### Natural killer (NK) cells

Apart from T cells and macrophages, little is known about the effects of KDs and KBs on other immune cells in the TME. However, some changes in the NK cell-mediated anti-tumor immune response have been reported. By recognizing tumor cells based on their lack of MHC-I expression, NK cells complement CD8+ T cells in the cytotoxic immune response to cancer cells [45].

Similar to cytotoxic T cells, NK cells exhibit glucose dependency in their activated state and require glycolysis to effectively slow tumor growth [46]. However, activated NK cells have been reported to survive under low glucose availability in the TME of multiple myeloma patients [47]. A study by Sheppard et al*.* revealed that fatty acids fuel NK cells, equipping them to fight malignant cells [48].

Accordingly, KDs resulted in elevated levels of NK cells in glioma- and colon cancer-bearing mice compared to standard diet-fed groups [13, 22]. Interestingly, qualitative but not quantitative KD-induced changes in NK cells, featuring higher IFN-γ and TNF-α production by NK cells, have been observed in mouse models of glioma and colon cancer [20, 24]. The impact of a KD on NK cells could at least partially be explained by a decrease in intratumoral lactate, alleviating the suppression of NK cell activity by lactate [21, 32]. Furthermore, the competition of NK cells with cancer cells for glucose could explain why a KD, supplying an alternative energy source via KBs, ameliorates an NK cell-mediated anti-tumor immune response. Gene expression analysis proved *OXCT1* expression in NK cells of healthy donors [36], indicating the ability of NK cells to use KBs for energy supply. However, knowledge on the capability of NK cells to metabolize KBs is lacking, requiring further investigation.

### Myeloid-derived suppressor cells (MDSCs)

MDSCs complement Tregs and M2 macrophages in the immune-suppressive and tumor-promoting niche of the TME, and are similarly associated with poor clinical outcomes in cancer patients. Targeting MDSCs in cancer therapy has therefore gained importance in recent years [49].

In the TME, MDSC proliferation and function are fostered by glycolysis and FAO, respectively [50]. Considering that fatty acids have been reported to promote the immune-suppressive activity of MDSCs [51], application of a KD might even support the immune-suppressive properties of MDSCs.

Yet, murine studies of glioblastoma, colon cancer, and pancreatic cancer reported a reduction of tumor-infiltrating MDSCs in KD-fed compared to standard diet-fed mice, helping to overcome immune evasion [13, 21, 22]. Since lactate is known to promote the development of MDSCs [21], a KD-associated decline of intratumoral lactate could explain the observed reduction of tumor-infiltrating MDSCs. Knowledge about KB metabolism in MDSCs is scarce, making the search for mechanisms underlying the decrease of MDSCs in the TME, other than lactate-deprivation, rather difficult. Wei et al*.*, however, demonstrated a decrease in cancer-associated fibroblasts (CAFs) accompanying reduced infiltration by MDSCs in colon cancer-bearing mice receiving a KD [22]. Considering that CAFs are involved in the recruitment of immune-suppressive cells, including MDSCs, to the TME [52], a KD-mediated reduction of CAFs could contribute to the lower number of tumor-infiltrating MDSCs.

In conclusion, the relationship between a KD and a possible anti-tumor immune response is exceedingly complex. Deciphering this interplay could pave the way for new treatment strategies. Further research will enable better understanding of the interaction between tumor, immune system and metabolism, as well as the effect of metabolic therapies on this interplay.

## KD as an adjunct metabolic therapy to support immune checkpoint blockade

The herein described studies, which strongly implicate a link between cancer immune surveillance and KD regimens, support further investigation of combined KD and ICB anticancer therapy. Although ICB substantially improves survival outcomes for various cancer entities, it still has various limitations, including metabolic barriers, which often prevent sustainable remission [53]. Metabolic barriers such as hypoxia, competition for nutrients, and high lactate levels in the TME pose obstacles to immunotherapy [54], but could potentially be targeted by a therapeutic KD regimen.

We know of four preclinical studies which have investigated the effect of a KD or BHB-supplemented standard diet as an adjunct to immunotherapy to overcome these hurdles. In prostate cancer [55], colon cancer [22, 25], melanoma, renal cancer, and lung cancer [33], KDs as well as BHB-supplemented standard diets synergized with ICB therapy to slow tumor growth and to improve survival, even in tumors resistant to ICB monotherapy [33, 55] (Table 1). The superior outcome of anti-PD-1 and anti-CTLA-4 therapy observed upon a concomitant administration of a KD was mimicked or even excelled by BHB supplementation in murine models of prostate, renal and lung cancers as well as melanoma [33, 55].

As outlined above, KDs dampen immunosuppression and boost the anti-tumor immune response in several tumor entities. Similarly, an increase in tumor-killing M1 macrophages, as well as quantitative and qualitative enhancement of CD8+ T cells, as indicated by increased IFN-γ, granzyme B, TNF-α, and IL-2 expression, was ascribed to the addition of a KD to an anti-PD-1 and/or anti-CTLA-4 treatment in prostate and colon cancers. Concomitantly, infiltration by pro-tumorigenic immune cells such as M2 macrophages, Tregs, and MDSCs was mitigated or not affected by a KD boost [25, 55]. Moreover, immunosuppression was further decreased by downregulation of PDL-1 on macrophages in the TME of melanoma-bearing mice and on colon cancer cells [25, 33]. Dai et al*.* explained this effect as being mediated by upregulation of AMP-activated protein kinase (AMPK) upon KD administration, since AMPK can initiate the degradation of PDL-1 [25]. Generally, the communicative networks in the TME were strengthened, as antigen-presenting pathways were upregulated in immune and tumor cells with combination KD and ICB therapy [25, 55].

Other mechanisms that may underlie the observed benefits include epigenetic alterations, i.e., BHB-mediated HDAC inhibition, since histone deacetylation poses a potential vulnerability to overcome resistance to immune therapy [56]. Of note, Murphy et al*.* reported that HDAC inhibition upregulated MHC-I expression on tumor cells, thereby enhancing their immunogenicity [55].

Furthermore, a KD-induced reduction of intratumoral lactate could support ICB. Lactate not only attenuates the function of cytotoxic immune cells while promoting immune-suppressive cells in the TME, it also decreases the efficacy of anti-PD-1 immunotherapy [57]. A KD could therefore alleviate these lactate-mediated barriers to ICB therapy success.

Overall, these studies evidently show that a KD can sensitize tumors, even those with initial resistance, to ICB therapy via a plethora of different mechanisms to slow tumor growth and prolong survival in murine models.

## Conclusions

Until now, blocking vital metabolic pathways to target cancer cells has been perceived to be rather challenging, because metabolically similar, activated, inflammatory immune cells will often be affected as well, hampering an effective anti-tumor immune response. A KD could help to overcome this limitation, as cytotoxic immune cells could be fueled by KBs, while immune-suppressive cells and cancer cells, largely unable to metabolize BHB, will perish due to nutrient deprivation.

**Fig. 1:**
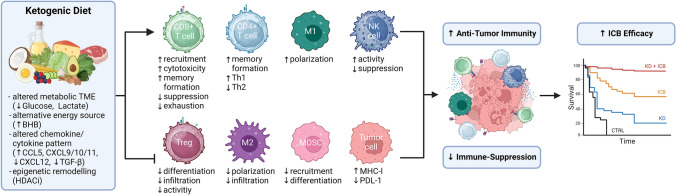
Proposed model of KD-induced reprogramming of an anti-tumor immune response: KD induces a decrease (↓) in glucose and lactate levels but an increase (↑) in ketone bodies, primarily beta-hydroxybutyrate (BHB). KD promotes anti-tumor immunity (positively affecting CD8+ T cells, CD4+ T cells, M1 macrophages, natural killer (NK) cells) and alleviates immune-suppression (negatively affecting regulatory T cells (Tregs), M2 macrophages, myeloid-derived suppressor cells (MDSC), tumor cells), leading to increased efficacy of immune checkpoint blockade (ICB) therapy. Note: CCL: C–C motif chemokine, CXCL: C-X-C motif chemokine, HDACi: histone deacetylase inhibition, IFN-γ: interferon gamma, MHC-I: major histocompatibility complex 1, PDL-1: programmed cell death ligand 1, TGF-β: transforming growth factor beta, TME: tumor microenvironment. (Created in BioRender. Stefan, V. (2024) BioRender.com/w68z545)

Accordingly, the studies we reviewed herein demonstrate that a KD remodels the TME, contributing to an eradication of cancer cells in preclinical tumor models (Fig. 1). Even though the most prominently reported immune-stimulatory effects of a KD are related to T cells and macrophages, effects on other immune cell populations are also suggested. It remains to be further elucidated how a KD impacts distinct subtypes of cells in the TME which until now have not been well-investigated, for example, B cells, CAFs, and dendritic cells. Moreover, further studies should aim to illuminate the relationship between metabolic and immunological changes in the TME to pinpoint KD-mediated mechanisms.

Owing to the above-described immune-modulatory activities of KDs, the dietary intervention was successful in sensitizing even resistant tumors to ICB therapy [33, 55]. The combination of a KD with ICB elicited a superior anti-tumor immune response, mitigated tumor growth, and prolonged survival in five different murine tumor entities (Table 1). Intriguingly, this combinatorial treatment regimen induced long-lasting, specific immunity and prevented tumor growth upon rechallenge in a mouse model of lung cancer [33]. Exploring whether and how a KD contributes to the establishment of long-lasting anti-tumor immune memory, and potentially aids in relapse prevention, will be crucial for determining its clinical relevance.

While preclinical studies of KD-ICB combinations are scarce, there are no reports from clinical studies so far. There is, however, evidence that a similar ketosis-inducing regimen, the so-called fasting-mimicking diet, mediates superior anti-tumor immune responses and synergizes with ICB in clinical trials [58–60]. Recently, recruitment for one of the first studies combining KD and ICB for the treatment of renal cell carcinoma, “KETOREIN”; was initiated (NCT05119010). Prospectively, in addition to ICB, CAR-T cell therapy has great potential to benefit from a KD, considering the stimulatory and the exhaustion-preventing effects of a KD on T cells.

In summary, KD has immune-modulatory effects that could boost an anti-tumor immune response, thus supporting the initiation of clinical studies combining ICB with a KD.

## Data Availability

No datasets were generated or analyzed during the current study.

## Abbreviations

AcAc: Acetoacetate
AMPK: AMP-activated protein kinase
BHB: Beta-hydroxybutyrate
CAF: Cancer-associated fibroblast
CCL: C–C motif chemokine
CTLA-4: Cytotoxic T lymphocyte-associated protein 4
CXCL: C-X-C motif chemokine
FAO: Fatty acid oxidation
HDAC: Histone deacetylase
ICB: Immune checkpoint blockade
IFN-γ: Interferon gamma
IL: Interleukin
Kbhb: Lysine beta-hydroxybutyrylation
KB: Ketone body
KD: Ketogenic diet
LAG-3: Lymphocyte-activation gene 3
MDSC: Myeloid-derived suppressor cell
MHC-I: Major histocompatibility complex 1
NK cell: Natural killer cell
OXCT1: 3-Oxoacid CoA-transferase 1
OXPHOS: Oxidative phosphorylation
PD-1: Programmed cell death protein 1
PDL-1: Programmed cell death ligand 1
SCOT: Succinyl-CoA-3-oxaloacid CoA transferase
TCA: Tricarboxylic acid
TGF-β: Transforming growth factor beta
TIL: Tumor-infiltrating lymphocyte
TME: Tumor microenvironment
TNF-α: Tumor necrosis factor alpha
Treg: Regulatory T cell
